# Clinical evaluation of multi-atlas based segmentation of lymph node regions in head and neck and prostate cancer patients

**DOI:** 10.1186/1748-717X-8-229

**Published:** 2013-10-03

**Authors:** Carl Sjöberg, Martin Lundmark, Christoffer Granberg, Silvia Johansson, Anders Ahnesjö, Anders Montelius

**Affiliations:** 1 Department of Radiology, Oncology and Radiation Sciences, Uppsala University, Uppsala SE-751 85, Sweden; 2 Elekta Instrument AB, Box 1704, Uppsala SE-751 47, Sweden; 3 Department of Medical Radiation Physics, Uppsala University Hospital, Uppsala SE-751 85, Sweden; 4 Department of Radiation Sciences, Radiation Physics, Umeå University, Umeå SE-901 87, Sweden

**Keywords:** Atlas-based segmentation, Radiotherapy, Head and neck, Prostate, Delineation time, Multi-Atlas segmentation

## Abstract

**Background:**

Semi-automated segmentation using deformable registration of selected atlas cases consisting of expert segmented patient images has been proposed to facilitate the delineation of lymph node regions for three-dimensional conformal and intensity-modulated radiotherapy planning of head and neck and prostate tumours. Our aim is to investigate if fusion of multiple atlases will lead to clinical workload reductions and more accurate segmentation proposals compared to the use of a single atlas segmentation, due to a more complete representation of the anatomical variations.

**Methods:**

Atlases for lymph node regions were constructed using 11 head and neck patients and 15 prostate patients based on published recommendations for segmentations. A commercial registration software (Velocity AI) was used to create individual segmentations through deformable registration. Ten head and neck patients, and ten prostate patients, all different from the atlas patients, were randomly chosen for the study from retrospective data. Each patient was first delineated three times, (a) manually by a radiation oncologist, (b) automatically using a single atlas segmentation proposal from a chosen atlas and (c) automatically by fusing the atlas proposals from all cases in the database using the probabilistic weighting fusion algorithm. In a subsequent step a radiation oncologist corrected the segmentation proposals achieved from step (b) and (c) without using the result from method (a) as reference. The time spent for editing the segmentations was recorded separately for each method and for each individual structure. Finally, the Dice Similarity Coefficient and the volume of the structures were used to evaluate the similarity between the structures delineated with the different methods.

**Results:**

For the single atlas method, the time reduction compared to manual segmentation was 29% and 23% for head and neck and pelvis lymph nodes, respectively, while editing the fused atlas proposal resulted in time reductions of 49% and 34%. The average volume of the fused atlas proposals was only 74% of the manual segmentation for the head and neck cases and 82% for the prostate cases due to a blurring effect from the fusion process. After editing of the proposals the resulting volume differences were no longer statistically significant, although a slight influence by the proposals could be noticed since the average edited volume was still slightly smaller than the manual segmentation, 9% and 5%, respectively.

**Conclusions:**

Segmentation based on fusion of multiple atlases reduces the time needed for delineation of lymph node regions compared to the use of a single atlas segmentation. Even though the time saving is large, the quality of the segmentation is maintained compared to manual segmentation.

## Background

In radiotherapy for local tumour control all anatomical structures of interest for dose quantification must be delineated before the treatment can start. Small structures with well-defined edges can be delineated manually in a short period of time without large inter-observer variation. Structures or organs such as lymph node regions that are hard to discriminate in CT images implies use of segmentation protocols based on indirect characteristics of nearby, visible anatomical structures [1,2]. Manual delineation of these structures can be very time-consuming, especially if they are large and irregularly shaped. Atlas based segmentation, where deformable registration is used to create delineations of regions of interest for a new image by transforming pre-made delineations of the corresponding structures in existing images [3], is assumed to reduce segmentation workload and time compared to manual segmentation under such conditions [4-6].

When the anatomy change of a patient between two image sets is small a single registration of an image set might be adequate to transform delineations that can be clinically approved with little or no editing. This can be the case for example if a patient is repeatedly imaged at different fractions of a radiotherapy treatment, or imaged by different modalities using similar patient setup procedure. For new patients where no previous delineated images exist, deformable registration to the new image using images from other patients as an atlas can be performed. In the latter case, minor or major editing of the resulting delineations is usually required to achieve a clinically acceptable result [4-6]. However, the required editing workload can be significantly shorter than to manually segment the new image from scratch.

In this work we will use the term “proposal” to denote a segmentation result before any manual editing is used to improve it. Several studies have shown that multi-atlas segmentation, where proposals from several different atlas patients are fused to yield a resulting proposal, can improve the result compared to the use of a single atlas [7-9]. We compare the segmentation time gains for editing of single atlas and multi-atlas segmentation proposals versus a manual segmentation. For multi-atlas fusion we use the recently introduced probabilistic weighting fusion algorithm (PWF) [10]. We focus on the target volume of lymph node regions for head and neck cancer (HNC) patients, and pelvic lymph node regions for prostate cancer patients. Besides work load analysis the similarity of the structures from the different methods are analysed using the Dice similarity coefficient and the volume of the structures.

## Methods

### Atlas material

The atlas database for the HNC cases consisted of image sets of 11 patients with different diagnoses in various stages randomly chosen from the clinical data. For use as a single atlas, one male and one female patient image set were subjectively selected by a radiation oncologist as being the most representative out of the available cases. The same male atlas was used for all male patients, and the same female atlas for all female patients. For all HNC atlas cases the lymph node regions of the patients were segmented in accordance with a generally adapted guideline for head and neck target and risk organ delineation [1].

For the prostate patients, an atlas database was established containing 15 prostate patient image sets randomly chosen from the clinical data. Again, a representative case with an anatomy that was least deformed by malignancies was selected for use as a single atlas. The pelvic lymph node regions of the prostate patients were delineated consistently following the protocol proposed by Taylor et al. [2].

### Patient material

For the HNC patients, two female and eight male patients not part of the atlas material were randomly selected from the available clinical data and used as test cases. Ten prostate cancer patients, also different from the patients used for the atlas database, with pelvic lymph node involvement were included in this study. The same segmentation guidelines as used for the atlas images were used for the manual segmentation of the test patient images. All delineations were created in a commercial treatment planning system (Oncentra 4.0).

### Deformable registration

**Figure 1:**
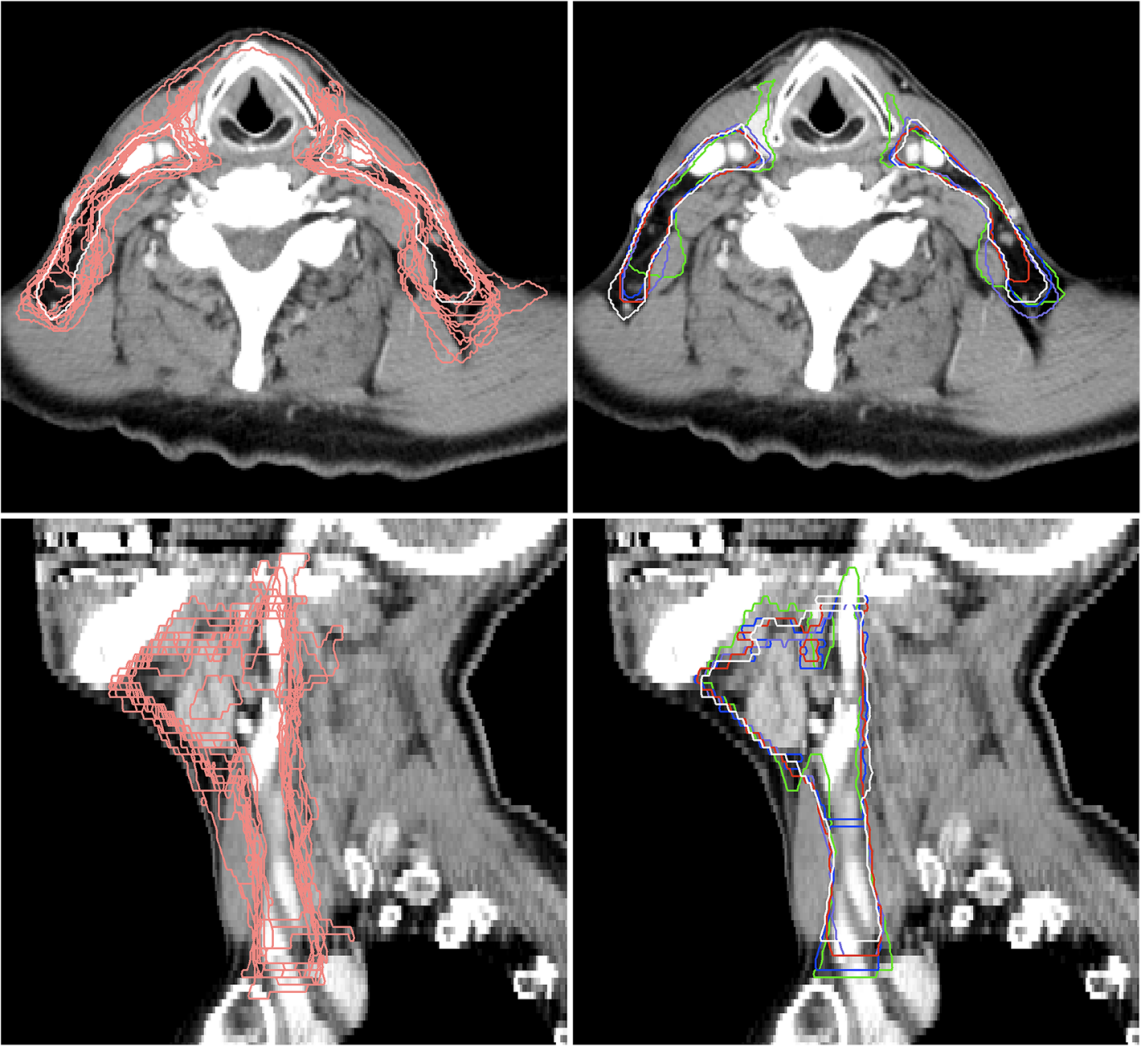
**Head and neck patient segmentation example.** Visualization of the different segmentations of one patient’s lymph node regions of the head and neck cases in a transversal slice (top) and a sagittal slice (bottom). The segmentations are: manual (white), individual 11 atlases (pink, shown in the left column), single atlas (green), PWF multi-atlas (purple), edited single atlas (blue) and edited fused atlas (red).

**Figure 2:**
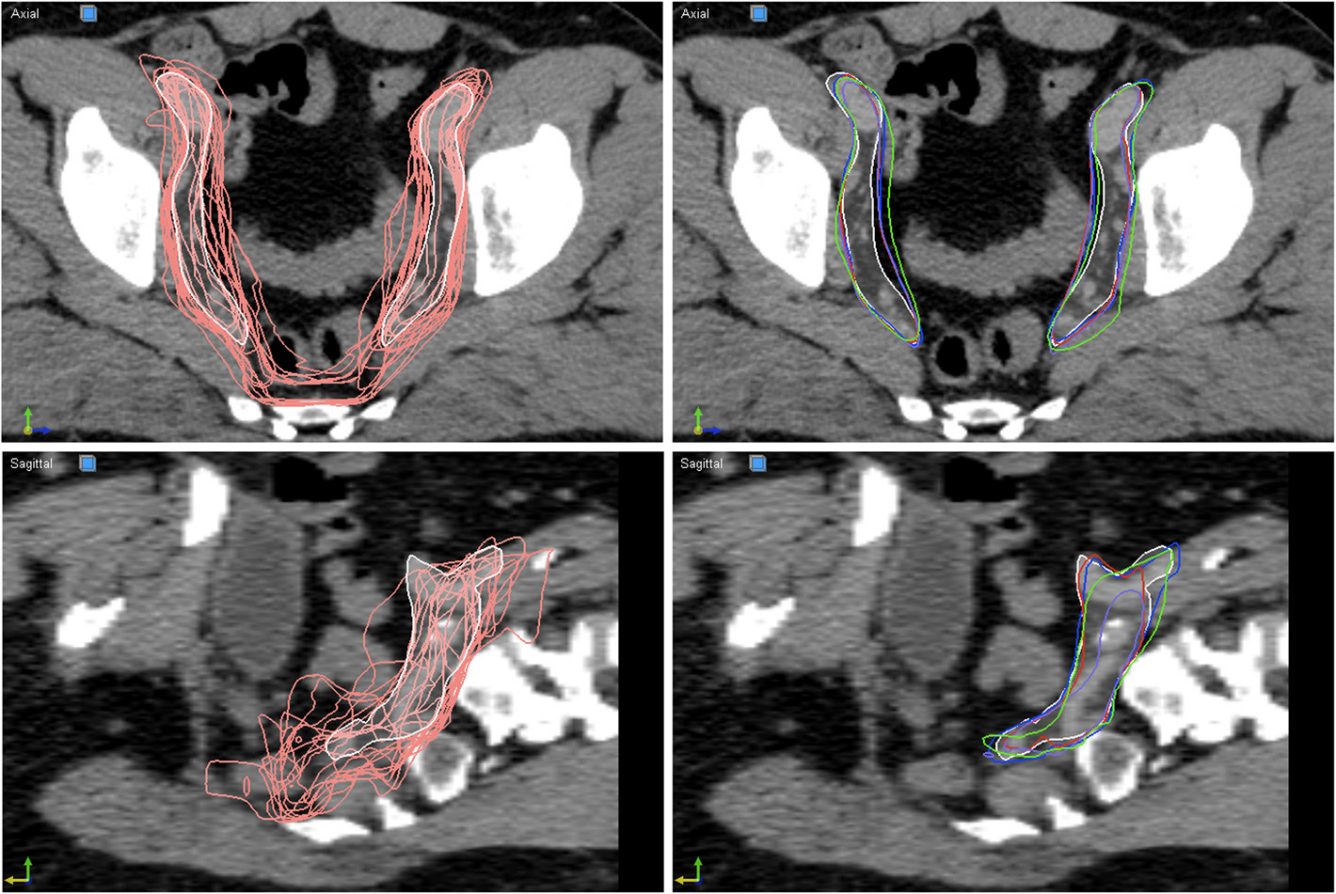
**Prostate patient segmentation example.** Visualization of the different segmentations of one patient’s lymph node regions for the prostate cases in a transversal slice (top) and a sagittal slice (bottom). The segmentations are: manual (white), individual 15 atlases (pink, shown in left column), single atlas (green), PWF multi-atlas (purple), edited single atlas (blue) and edited fused atlas (red).

A commercial registration software (VelocityAI) was used for creating the individual atlas results. Starting from an initial translation given by the user, the software first determines a linear registration using translation, rotation and isotropic scaling. It then links a deformable registration to the linear transform by optimizing B-spline coefficients [11] with regard to an image similarity measure. As a result of the deformable registration, a total of *N* deformed lymph node segmentation proposals for each patient were available, where *N* = 11 for the HNC patients and *N* = 15 for the prostate patients. Example segmentation proposals from the registration software can be seen in the left panel of Figure 1 for one HNC patient and Figure 2 for one prostate patient, together with the manual segmentation.

### Probabilistic weighting fusion

All atlas segmentations were exported from VelocityAI and imported into an in-house developed implementation of the probabilistic weighting fusion algorithm [10]. The resulting fused proposal from this multi-atlas application is a weighted mean shape using weights based on the local registration success, as measured by an image similarity measure. The normalized cross correlation was used as image similarity measure, calculated for a volume consisting of the deformed structure including a uniform dilation margin of 10 mm for the head and neck patients and 50 mm for the prostate patients. The PWF method uses as input parameter the ratio *k*/*s*, where *k* is the proportionality of segmentation quality to image registration quality, and *s* is the expected spread of segmentation quality. The fused segmentations are constructed by weighted superposition of distance maps with weights calculated from the *k*/*s* parameter according to section 2.5 of [10]. For optimal performance *k*/*s* should be optimized from a large material. However, in lack of such data, preliminary testing indicated that *k*/*s* could be set to 0.5 for the head and neck patients and to 20 for the prostate patients. The lower limit of *k*/*s* is zero, which is equivalent to use of equal, unbiased weights, while an infinitely high value of *k*/*s* results in selection of the most similar registration as output.

### Segmentation editing

For each test patient, both the single atlas proposal and the fused multi-atlas proposal were imported into the treatment planning system where all editing of the delineations were performed. This assured use of identical editing tools, familiar to the radiation oncologist and independent of registration method. The same radiation oncologist performed all delineations as well as all segmentation editing. The structures delineated from scratch and used as reference were created using a “freehand drawing tool” and a “polygon drawing tool”. For editing of the atlas based proposals mainly the “pearl drawing tool” was used that provides a circular brush (“pearl”) of variable diameter which can be moved to “push” the contour lines to enlarge or diminish the segmented region. This tool was found to be particularly effective for adjusting smoothly curved contour lines. The atlas proposals and the edited segmentations can be seen in the right panels of Figures 1 and 2.

### Time scoring

The main objective for this study was to investigate how much time the radiation oncologist potentially could save by using atlas based segmentation tools. For this purpose, the radiation oncologist recorded the manual contouring time as well as the time needed for editing of each individual structure for every patient. To reduce bias from editing the same structure multiple times, a minimum of one week was spent between each of the three segmentation occasions for a patient. Also, the different segmentation editing options were performed in varying order. The auxiliary time for studying medical records, MRI and PET images, etc., was omitted from the scored time.

### Evaluation of structure volumes and overlap

The commonly used Dice Similarity Coefficient (DSC) [12] was used for evaluation of segmentation quality, and to quantify the similarity between different segmentations of a structure. DSC is a measure of the spatial overlap between structures ranging from 0 to 1, where 0 means no overlap and 1 equals a perfect match. The DSC was calculated for the clinical data based on the number of voxels contained in every structure. A voxel was deemed to be inside the structure if the voxel centre was located inside the structure.

A second evaluation tool was to determine the volumes of the segmented structures. The volumes could be extracted from both the treatment planning system and the registration software but, since the two systems gave slightly different results due to different handling of partial voxel volumes, the volumes from the registration software only were used to ensure consistency.

## Results

### Timesaving

**Figure 3:**
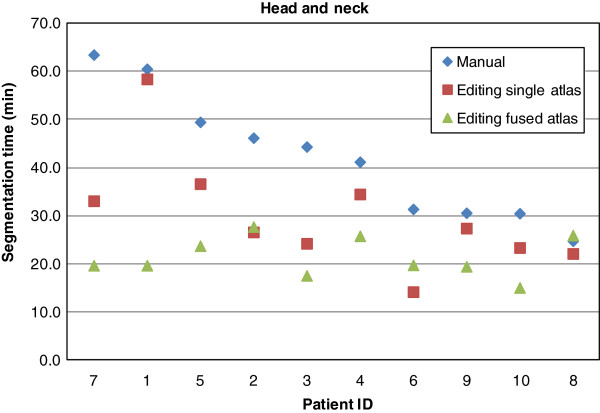
**Head and neck patients segmentation times.** Segmentation times for the individual Head and neck patients. The order of the patient IDs is sorted by decreasing manual segmentation time.

**Figure 4:**
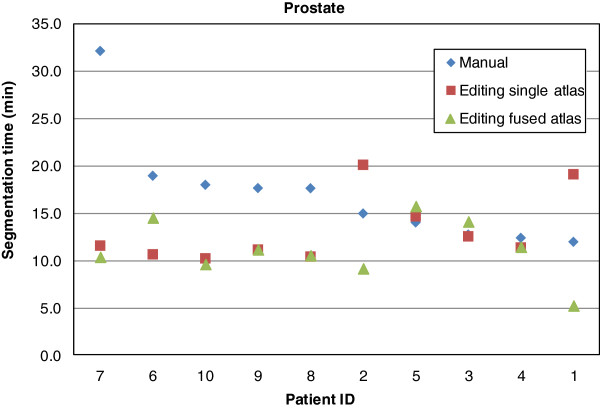
**Prostate patients segmentation times.** Segmentation times for the individual prostate patients. The order of the patient IDs is sorted by decreasing manual segmentation time.

The average manual segmentation times and editing times of single atlas and fused atlas proposals are presented in Table 1, and the time gains are shown in Table 2. These results demonstrate that using both single atlas and fused atlas methods yield on average significant time-savings, and that the fused atlas method is superior to the single atlas method. Figures 3 and 4 displays plots of segmentation times for the individual patients. The fused atlas method yields consistently lower segmentation times compared to manual segmentation and single atlas segmentation. This effect is seen more clearly for the head and neck lymph node segmentations, which have less well defined boundaries than the prostate lymph node segmentations.

**Table 1:** Segmentation time

	***Manual***	***Editing single atlas***	***Editing fused atlas***
**Head & Neck**	42.3 (13.2)	30.1 (12.0)	21.4 (4.1)
**Prostate**	17.1 (5.9)	13.2 (3.6)	11.2 (3.0)

Average lymph node segmentation times in minutes with the spread (one st.d.) shown within parenthesis. For atlas based segmentation, the editing time to correct the resulting proposal is shown.

**Table 2:** Segmentation time gain

	***Editing single atlas vs. manual***	***Editing fused atlas vs. manual***	***Editing fused atlas vs. editing single atlas***
**Head & Neck**	29%*, p = 0.044	49%*, p = 1.5e-4	31%*, p = 0.045
**Prostate**	23%	34%*, p = 0.012	15%

Relative gain in lymph node segmentation time. The editing time of atlas proposals are compared to manual segmentation times, and editing times for fused atlas proposals are compared to editing times for single atlas proposals. Improvements marked with * are statistically significant at the 0.05 level for a 2-sided t-test.

### Volume ratios

The volumes of the segmented structures are shown in Table 3 as ratios compared to the manual segmentations. The volume of the single atlas proposal does not differ significantly from the manual segmentation for the head and neck case, while the prostate case is on average 26% larger. This is most likely due to a large lymph node region for the selected single case used as atlas compared to the patient material and demonstrates the difficulties of selecting a generic atlas, valid for single atlas segmentation for a large patient population. The fused atlas proposals are significantly smaller (26% for head and neck and 18% for prostate cases) than that of the manual segmentation, a consequence of the fusion process which emphasis the central part of the volume common to most of the proposals, on the behalf of the more peripheral parts. Hence, most of the corrective editing of multi-atlas proposals consists of enlarging the proposed structures. It can also be seen that there is a tendency that the size of the proposed structure can influence the editing. This has resulted in an edited fused atlas segmentation that on average is 9% smaller than the manually segmented structure for head and neck cases. Similarly an edited single atlas proposal is 3% larger than the corresponding manually segmented structure.

**Table 3:** Volume ratio

	***Single atlas***	***Edited single atlas***	***Fused atlas***	***Edited fused atlas***
**Head & Neck**	1.04	1.03	0.74*, p = 0.047	0.91
**Prostate**	1.26*, p = 0.026	1.06	0.82*, p = 0.019	0.95

Volume ratios for atlas based segmentation proposals of lymph node levels compared to manual delineations. Ratios marked with * are statistically significant at the 0.05 level for a 2-sided t-test.

### Dice similarity coefficient

**Figure 5:**
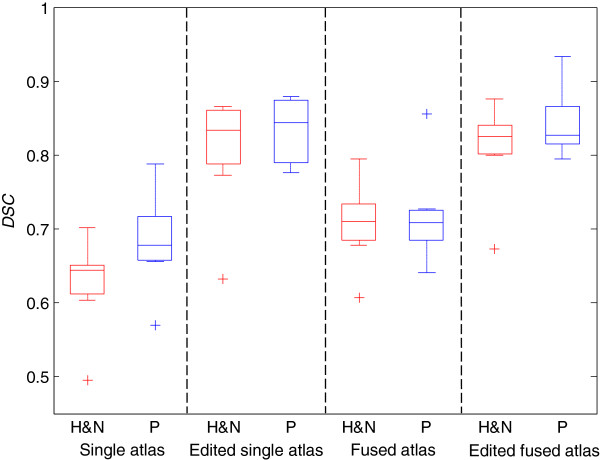
**Dice similarity coefficients.** Box plot with DSC values for the lymph node regions (median, quartiles, outliers). Red boxes corresponds to head and neck cases (H&N) and blue boxes to prostate cases (P).

Figure 5 displays a box plot of the DSC values for the 10 patients for both un-edited and edited proposals for single and fused atlases, compared to the manual segmentations. The fused multi-atlas atlas proposal seems to yield a somewhat higher DSC value compared to the single atlas proposal and for the HNC cases the difference is significant for a Wilcoxon rank sum test (p = 0.0036). After editing, both proposals reach approximately the same median value (0.82-0.84) for the head and neck as well as the prostate lymph node regions, indicating an upper bound due to intra-operator variability.

## Discussion

In this work, one single atlas was selected per anatomical site and sex. If instead this single atlas would be selected manually for each registration, a better registration result might be possible. To select this atlas quickly might however be difficult. Another problem with this approach is that since this single atlas might be more similar in some areas than others, segmentation similarities will still probably be lower than using a fused atlas method.

Of interest is to note that the volume of the fused atlas proposal was on average smaller than the single atlas proposal. The pearl editing tool was regarded by the radiation oncologist as somewhat easier to use when starting with a small volume where the borders are pushed outwards to the desired position rather than the opposite, which would mean that smaller proposals would be preferred over larger.

The segmentation time reduction was largest for the head and neck lymph nodes. This is most likely due to the complexity and individual variation for these structures. The pelvic lymph nodes are closely linked to the neighbouring bone structures. This facilitates both manual segmentation and editing, leading to a smaller reduction of segmentation times than for the head and neck nodes. However, even if the magnitude of time saved per patient for the pelvic lymph nodes is modest, for a large throughput of patients the gain can still be of importance.

The accuracy measures showed that on average most atlas based proposals were reasonable but no segmentation proposal was approved by the radiation oncologist without any further editing. Thus, fully automated segmentation may still not be feasible. Volume measures and DSC values together gave a good picture of the segmentation accuracy results, which was confirmed in visual inspections.

## Conclusions

Atlas based segmentations of lymph node regions for prostate and head and neck patients significantly saves time, on average, for the radiation oncologists compared to manually segmenting each patient. This is demonstrated even when segmentation proposals need to be extensively edited. Fused atlas proposals are generally superior to single atlas proposals, both as measured by a reduction in segmentation time and as measured by a higher binary overlap.

## Competing interests

Carl Sjöberg is an employee of Elekta Instrument, AB. This work was funded by Nucletron, Landstinget i Uppsala län, Cancerfonden and Elekta Instrument AB.

## Authors’ contributions

Please see sample text in the instructions for authors. CS: Developed model for fusion of multiple atlases. Main author of paper CG: Responsible for acquisition and analysis of prostate patient data. Revision of paper ML: Responsible for acquisition and analysis of head & neck patient data. Revision of paper SJ: Responsible for acquisition of patient material and target delineations. Revision of paper AA: Model development and drafting of manuscript AM: Design of study, drafting of manuscript. All authors read and approved the final manuscript.
